# Functional Toxicogenomics: Mechanism-Centered Toxicology

**DOI:** 10.3390/ijms11124796

**Published:** 2010-11-24

**Authors:** Matthew North, Chris D. Vulpe

**Affiliations:** Department of Nutritional Science and Toxicology, University of California Berkeley, Berkeley, California 94720, USA; E-Mail: mnorth@berkeley.edu

**Keywords:** toxicity testing, functional toxicogenomics, toxicity pathways, barcoding, yeast

## Abstract

Traditional toxicity testing using animal models is slow, low capacity, expensive and assesses a limited number of endpoints. Such approaches are inadequate to deal with the increasingly large number of compounds found in the environment for which there are no toxicity data. Mechanism-centered high-throughput testing represents an alternative approach to meet this pressing need but is limited by our current understanding of toxicity pathways. Functional toxicogenomics, the global study of the biological function of genes on the modulation of the toxic effect of a compound, can play an important role in identifying the essential cellular components and pathways involved in toxicity response. The combination of the identification of fundamental toxicity pathways and mechanism-centered targeted assays represents an integrated approach to advance molecular toxicology to meet the challenges of toxicity testing in the 21^st^ century.

## 1. Introduction

Traditional toxicity testing approaches are inadequate to meet the challenge of current toxicity assessment requirements. Tens of thousands of chemicals are used annually in industry that have no toxicological data associated with them, and this number is ever-increasing [1]. In addition, recent advances such as nanotechnology have introduced new classes of compounds into general use that represent an additional challenge to risk assessment. Recent initiatives in policy and new developments in toxicology testing are providing the impetus and the means to address this lack of knowledge. For example, the recent European Community Regulation REACH (registration, evaluation, authorization and restriction of chemicals) will require the registration of ∼30,000 chemical substances over the next 11 years [2], with a primary aim to progressively substitute the most dangerous chemicals with suitable alternatives. Current toxicity testing of chemical compounds is based on extensive animal testing, which is time-consuming, expensive and unfeasible for this number of compounds. In addition, there is a continuing ethical imperative to reduce the amount of animal testing through the 3R’s (“reducing, refining, and replacing”) [3]. Alternative high-throughput approaches are clearly needed to meet this need. Animal toxicity tests are also limited in their ability to detect toxicity. Only a very limited subset of potential modes of action, as made clear by identification of the endocrine disruption activity of some chemicals, is generally assessed. Comprehensive mechanistic approaches are needed to assess the diversity of possible modes of toxicity. Functional toxicogenomics provides an important tool to identify critical pathways involved in toxicity.

## 2. *In Vitro* Assessment of Toxicity Pathways

Cell-based high-throughput screens are one aspect of this new mechanistic approach to toxicity testing. The number of potentially toxic compounds produced and used both in manufacturing and in the pharmaceutical industry requires that new methods be employed to accelerate toxicity testing. The changing nature of toxicity testing is highlighted in a 2007 report from the United States National Research Council (NRC), entitled *Toxicity Testing in the 21^st^ Century: A Vision and a Strategy* [4], which outlines a plan to modernize human health toxicity assessment based on the utilization of mechanistically-based high-throughput cellular assays [5]. Tox21, a collaborative effort between the National Institute of Environmental Health Sciences (NIEHS), the National Human Genome Research Institute (NHGRI), the U.S. Environmental Protection Agency (EPA) and recently the U.S. Food and Drug Administration (FDA), was established to respond to the NRC challenge to advance the state of toxicity testing. The premise of Tox21 is that human harm from chemicals can be inferred from activation of toxicity pathways in cells [6]. Toxicity pathways are defined by the NRC as “cellular response pathways that, when sufficiently perturbed in an intact animal, are expected to result in adverse health effects” [4]. The report did not identify specific toxicity pathways, but a recent review has argued that assessment of certain stress response pathways such as oxidative stress, heat shock, DNA damage, and endoplasmic reticulum (ER) stress response could be used in cell-based toxicological screening [7]. Implementation of a toxicity pathway approach to screening is facilitated by the availability of a wide variety of cellular assays developed by academic and commercial laboratories for many of these proposed mechanistic endpoints. In addition, many of these assays have been adapted for high-throughput screens. For example, the National Institutes of Health Chemical Genomics Center (NCGC) is currently screening thousands of compounds as part of Tox21 by assessing a wide variety of mechanistic endpoints [8]. Similarly, the EPA’s ToxCast program, started in 2006, aims to advance environmental testing by developing methods of prioritizing chemicals for further screening and testing to assist EPA programs in the management and regulation of environmental contaminants [9]. Phase I of ToxCast has screened a library of 309 chemicals, using 467 assays, with promising early results [10]. While these *in vitro* data will provide valuable mechanistic insights into the mode of action of potential toxicant compounds, they are limited to existing assays of known endpoints. Comprehensive assessment of toxicity will require identification of toxicity pathways and development of targeted assays to systematically assess potential modes of action.

## 3. Toxicogenomics: Omic Tools Applied to Toxicology

Toxicogenomics can provide insight into the mode of action of toxicants and allow for development of targeted cellular assays [11]. Toxicogenomics was defined as “the application of global mRNA, protein and metabolite analysis related-technologies to study the effects of hazards on organisms” [12]. The underlying premise of toxicogenomics is that a global assessment of the biology of chemical exposure can lead to a more thorough understanding of the mechanism of action of toxicants [13]. Toxicogenomics studies the interactions between the genome and adverse biological effects caused by exogenous agents such as environmental stressors, toxins, drugs and chemicals [14].

Toxicogenomics initially arose through the use of microarrays to assess global gene regulation (measured by relative abundance of mRNA) following treatment with various stressors (reviewed in [15]). One of the aims was to develop “fingerprints” of gene expression changes in response to treatment with different classes of known toxicants (oxidant stressors, polycyclic aromatic hydrocarbons *etc.*), which could then be used to gain insight into the mode of action of unknown compounds. Expression profiling has been widely used (across many organisms) to discover biomarkers for a wide range of toxicants (reviewed in [12–14,16,17]). The data obtained from expression profiling can be used to inform selection of mechanism-based assays, such as the NGCG assays discussed in the previous section, and thus are an important starting point to the identification of toxicity endpoints for cellular assays. However, interpretation and integration of toxicogenomics data from a wide range of sources and subsequent mining of these datasets remains a major challenge. Currently, several large-scale projects to collect toxicogenomic data and produce minable databases are underway, including the Japanese Toxicogenomics Project (TGP) [18], the European Innomed PredTox [19], and the Liver Toxicity Biomarker Study [20]. Similarly, other “omics” methodologies (e.g., proteomics, metabolomics) are being used to probe toxicity mechanisms and have been reviewed extensively elsewhere [12,14,21]. While useful, a limitation is that these omic technologies are correlative and do not determine the functional requirement of a gene for the cellular response to a toxicant. As a result, there has been extensive discussion of the need for quantifiable “phenotypic anchors”, in order to link the patterns of altered gene/protein/metabolite expression to specific parameters of well-defined indices of toxicity [22]. Functional toxicogenomics, in contrast to other omic approaches, can provide a direct link between gene and toxicant.

Functional genomics was defined as “the development and application of global (genome-wide or system-wide) experimental approaches to assess gene function by making use of information and reagents provided by physical mapping and sequencing of genomes” [23]. Functional genomics directly measures phenotype, and thus provides a direct link between a specific gene and the requirement for that gene product in the cellular response to treatment with a compound [24]. This functional information is obtained by screening collections of cells/organisms that lack either genes (through deletion) or proteins (through blocking translation by using technologies such as RNAi). While conceivably any phenotype could be measured, the viability or fitness of cells or organisms are frequently assessed as indicators of alterations in the response to a compound. Recently, the capacity of functional genomics to provide an increased mechanistic understanding of toxicant-induced phenotypes [12] was recognized and consequently termed functional toxicogenomics.

## 4. Functional Toxicogenomics in Yeast

**Figure 1.**
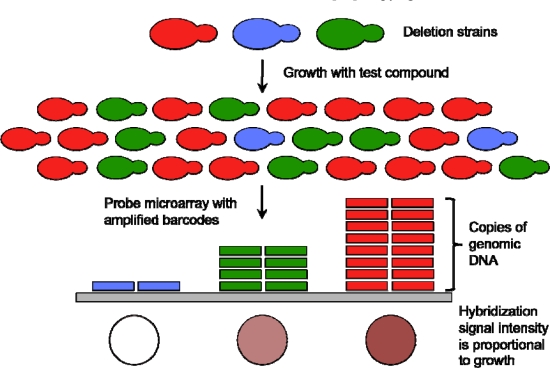
**Schematic representation of parallel deletion analysis (PDA).** PDA quantifies the relative abundance of each deletion strain in the pool. Adapted by permission from Macmillan Publishers Ltd: Nature Reviews Genetics [28], copyright 2001.

Functional toxicogenomics is functional genomics in the context of toxicology, *i.e.*, the study of the requirement for the biological activities of genes and proteins in the response of, and effect on, an organism by a toxicant [12]. In the budding yeast *Saccharomyces cerevisiae*, global (genome-wide) analysis of gene function was initially attempted using different applications of random mutagenesis to abolish gene function [25–27]. The sequencing of *S. cerevisiae*, the first eukaryote genome to be sequenced, allowed a directed all-inclusive approach to be taken. All identified genes were systematically deleted through a PCR-based approach, using unique 20 bp “barcodes” to disrupt ORFs and creating a set of “knockout” yeast strains for both non-essential and essential genes. An important consequence of “molecular barcoding” is that multiple deletion strains can be pooled and assayed for growth simultaneously (Figure 1).

Parallel deletion analysis (PDA) is a powerful technique as it allows for quantitative analysis of the fitness of every deletion strain tested simultaneously (the so-called “deletome” [29]). In this approach, the molecular barcode tags from all strains present in a pooled culture can be amplified simultaneously in a single PCR reaction (using a pair of PCR primers that anneal to the common regions flanking the inserted barcodes that are present in all strains). These amplified tags can be hybridized to microarrays containing oligonucleotides complementary to the barcodes, and the resulting hybridization signal is proportional to the number of cells of that strain present when the pooled culture was sampled By comparing the hybridization signal of a strain from a culture that has been exposed to a suspected or known toxicant (“treatment”) to the signal from the same strain from an untreated culture (“control”), a “fitness score” can be derived, and deletion strains whose growth is significantly influenced by the treatment compound can be determined [30,31]. If a deletion strain is significantly sensitive to treatment, the gene product absent in that strain may be a cellular target of, or may be involved in the cellular response to, that treatment compound. The technical aspects of PDA have been described in greater detail elsewhere [32–34].

This approach was initially used with a small number of deletion strains as a proof of principle [30], but was soon expanded to include deletions representing more than one-third of the genome (2026 ORFs) [35]. This work identified many essential genes (*i.e.*, genes whose deletion cannot be tolerated), and an updated disruption method created two barcodes for some ORFs, an UPTAG and a DOWNTAG, which can be amplified independently (as opposed to the original single tag). There was shown to be good correlation between the two tags [35], and so this feature was adopted in the construction of the deletion strain collections currently in use [36]. The 2002 study by Giaever *et al* constructed four different yeast knockout (YKO) collections of strains: one collection of homozygous diploid deletions of all non-essential genes (4,757 strains), one of heterozygous diploids (5,916–essential and non-essential), and haploid collections of both mating types (4,815 *MAT***a**, 4,803 *MAT*α strains). This study also replaced the printed oligonucleotide arrays used previously with a commercially produced custom Affymetrix GeneChip, termed the TAG3 array. This array contained oligonucleotides complementary to the UP and DOWNTAG barcodes of every deletion strain. The TAG3 arrays were subsequently updated and replaced by TAG4, the array platform currently in use [37]. The TAG4 array contains five duplicate sets of probes (replicate features) and data from TAG4 arrays has been shown to be more reproducible and more accurate when compared with TAG3 [37]. To date, functional profiling using the YKO collections and the Affymetrix TAG arrays have been used in a number of studies to gain insight into the genetic requirements of the response to several compounds and nutritional states (Table 1). The choice of knockout collection used for profiling depends on the specific aims of the study being conducted. PDA using the heterozygous collection of essential deletions (Haplo Insufficiency Profiling–HIP) can reveal genomic profiles for the cellular targets of a drug or toxicant (reviewed in [38]), whereas use of the homozygous collection of non-essential deletions (HOmozygous deletion Profiling - HOP) can identify genes and pathways that buffer the drug or toxicant target pathway [39].

The development of high-throughput sequencing technologies has allowed a different method of barcode quantification. Termed “Bar-seq” (for Barcode analysis by Sequencing) this analysis relies on sequencing of the total amplified barcodes from a pool, rather than detection using a microarray. Initial results from Bar-Seq indicate that this approach is superior to microarrays in both dynamic range and throughput [40], and it is likely that as sequencing technologies become more affordable, they will replace microarrays as the technique of choice for PDA.

A major contribution of the yeast system has been to provide quantitative phenotypic data to validate microarray methods and data modeling procedures [55]. Interestingly, studies in yeast have revealed a low correlation between the regulation of a gene’s transcription and its requirement for growth under selective conditions [36,56], further suggesting that growth studies such as PDA are a better assay to identify genes required for the response to toxicant treatment [53]. However, data from expression and functional studies can be integrated to give a greater understanding of the adverse effects of toxicant exposure or an altered nutritional state [52].

*S. cerevisiae* is a good model for human and higher eukaryote disease and toxicity testing, as yeast has functional orthologs of many human disease genes [41,57,58]. Data from fitness screening in yeast is therefore informative in the context of the human response to a toxicant, in the cases where there is a human ortholog of identified candidate yeast genes whose deletion confers alters sensitivity to a compound. PDA has been applied to a number of toxicants, including arsenical compounds [53]. In one example, the human ortholog of a gene indentified in the arsenical screen as being required for resistance to arsenite in yeast was shown to be required for the resistance of human cells to arsenical compounds [59], providing evidence that genes identified by functional profiling in yeast can have homologous requirements in human cells.

The quantitative fitness data from PDA allow for bioinformatic analysis not possible with data from qualitative phenotypic screening approaches, such as spotting of strains to microtiter plates and assessment of growth by cell density. Network analysis software such as Cytoscape [60] can be used to map the fitness data from profiling studies onto a network constructed using known genetic and physical interactions between genes and proteins. Analytical tools such as the jActiveModules plug-in [61] can then be used to identify functional modules or sub-networks based on fitness data that may identify cellular processes or protein complexes affected by toxicant treatment. The extensive study of *S. cerevisiae* has resulted in excellent bioinformatic tools, such as Gene Ontology (GO) annotation that, through the use of web-based resources such as FunSpec [62], can be used to identify statistically over-represented GO categories within groups of genes (such as those identified as being required for tolerance of a toxicant). Human orthologs of yeast genes can be identified using databases such as YOGY (eukarYotic OrtholoGY), a powerful database that integrates data to allow identification of orthologs across species [63]. These bioinformatic approaches allow identification of functional “fingerprints” for compounds, much like expression profiling. Comparison of functional profiles between compounds can identify cellular responses and toxicity pathways that are common to multiple classes of toxicants.

A further concept that resulted from functional profiling experiments in yeast has been referred to as “combination chemical genetics” or “chemical genomics”. This is defined as “the systematic application of multiple chemical or mixed chemical and genetic perturbations, both to gain insight into biological systems and to facilitate medical discoveries” [64], and has been covered in a number of reviews [33,64–66].

While primarily concerned with identifying drug targets and undertaking mode of action determination, the tools developed for chemical genomics have potential applications in toxicology. These tools include novel yeast strain collections, such as the DAmP (decreased abundance by mRNA perturbation) collection, which contains hypomorphic alleles of genes (both essential and non-essential) [67–69]. The DAmP collection complements the heterozygous deletion collection and in some aspects has proven to be more sensitive, identifying several drug targets missed by the HIP assay [69]. Another exciting functional technology that may have applications in toxicology is the molecular barcoded yeast open reading frame (MoBY-ORF) library [70]. This is a library of barcoded vectors, each containing a single yeast gene under the control of its native promoter. There are numerous potential applications for this vector library, and so far it has been used in a complementation assay to identify genes that when mutated confer drug resistance to cells [70]. Data from assays using these various resources will eventually be integrated to improve sensitivity and data quality [71], and these approaches could be used in combination to aid the screening of potentially toxic compounds. Recent work undertaken to uncover interactions between genes through synthetic lethality (by generating libraries of double mutants) has produced extensive interaction networks [72], which may help to elucidate toxicity pathways in yeast and help to determine the mode of action of test compounds when integrated with data from functional screens.

Although functional screening in yeast is a powerful tool for helping to establish the toxicity of compounds, and for identification of conserved cellular processes required for sensitivity or tolerance to tested compounds, there are limitations of the yeast system. As a eukaryote *S. cerevisiae* is an excellent model organism for studying fundamental cellular processes but it is not an accurate indicator for determination of the toxic dose of a compound for human exposure, as in general *S. cerevisiae* is able to tolerate much higher doses of toxicants than human cells [53]. There are however other deficiencies with the yeast system. Clearly yeast cannot provide data on organ or tissue-specific toxicity and being unicellular, no insight into the roles of cell-cell signaling, such as endocrine disruption, by a compound can be deduced using this system. The great evolutionary distance between yeast and humans is also an issue. Although many yeast genes have functional orthologs in humans, many yeast genes have human homologs that encode proteins whose function is not conserved. Conversely there may be human orthologs of candidate yeast genes that cannot be determined using bioinformatics due to excessive sequence divergence of either the gene or protein encoded. A similar confounding factor that hinders the identification of human orthologs of yeast susceptibility genes is the presence in humans (and other higher eukaryotes) of gene families. Due to this, single yeast genes can have a large number of equally probable human orthologs, and it is at present impossible to predict which (if any) are the true human ortholog(s) of the yeast gene without experimentation. In order to address these issues, similar functional toxicogenomics technologies are now being developed in higher eukaryotic systems.

## 5. Functional Toxicogenomics in Higher Eukaryotes

Development of functional genomics in higher eukaryotic systems has been more challenging than in yeast, primarily due to the difficulty of creating homozygous gene knockouts in mammalian cells. Although techniques for gene disruption in mammalian cells have advanced, it is still time-consuming and expensive, and so creating a library of deletion cell lines similar to the yeast collections is not practical at this time. Small collections of targeted knockout cell lines have been produced in chicken DT40 cells, and are being used to study compounds such as formaldehyde [73], but there are no such targeted deletions yet available for mammalian cell lines.

Functional genomics in mammalian systems has been made possible by RNA interference (RNAi). RNAi works by targeting the expressed mRNA of a gene and preventing translation, effectively “knocking down” the function of the targeted gene. RNAi was first discovered in the nematode *Caenorhabditis elegans* [74], and has since been developed for use in many other organisms. The application of genome-wide RNAi has become an integral functional genomic tool for drug target identification and validation, pathway analysis and drug discovery [75], and can readily be applied to toxicology. Loss-of-function genetic screens using RNAi in human cell lines can be carried out in a high-throughput manner and, as a consequence of the functional nature of the approach, can lead to the identification of causal factors, as in yeast [76]. High-throughput RNAi (HT-RNAi) screening can be conducted using two different methods of RNAi, either using short hairpin RNA (shRNA) vector libraries (reviewed in [76]), or using chemically or enzymatically generated small interference RNAs (siRNA-reviewed in [75]).

shRNA screening (referred to as shRNA bar coding) uses vector libraries such as The RNAi Consortium (TRC) lentiviral library, which contains shRNAs targeting 17,200 humans genes [77]. Each shRNA is identifiable by a unique bar code, much like the yeast deletion collections. The bar code in shRNA libraries can be a separate DNA sequence in the plasmid vector, or it can be part of the shRNA cassette. shRNA bar code screening allows identification of shRNAs that give rise to a specific phenotype under the conditions of toxicant treatment. Cells from the cell line of choice are infected in sufficient numbers to ensure that the whole vector library is represented. Two replicate populations (pools) of cells are created, one untreated control and one pool treated with the test compound. Cells are then grown in petri dishes, and bar codes are recovered by PCR. Bar codes can then be hybridized to microarrays, similar to the yeast system, in order to quantify the abundance of shRNAs in both the control and treatment populations. Relative abundance of shRNAs can then be determined to identify those that are affected by the treatment compound. shRNA bar coding is very effective at identifying genes whose knockdown increases fitness under test conditions, and has been successfully applied to find genes whose inactivation play a role in cancer progression (reviewed in [76]). The detection of a decrease in shRNAs (equivalent to identifying sensitive yeast deletion strains) is more challenging, but has recently been achieved [78]. It is also possible to conduct shRNA screens using vectors that do not contain bar codes. In this case, shRNAs are recovered from a population of cells resistant to test compound treatment and are sequenced to identify the knocked down gene(s) [79].

siRNA cannot be used in this manner. In siRNA screening, cells are grown in microplates and a single siRNA is added to each well (single well screening). Cells can then be assayed using a variety of methods, including high content microscopy or viability screening, or through a number of different reporter assays (reviewed in [80]).

As previously mentioned, RNAi has been used extensively in other eukaryotes. RNAi screening is perhaps best characterized in *C. elegans* (reviewed in [81]) and has been used in toxicological studies [82]. It is also used extensively in the fruit fly *Drosophila melanogaster* (reviewed in [83]). In addition, RNAi screening is now possible in zebrafish, and has the potential to be extremely useful in environmental toxicity testing [84]. An advantage of these whole organism systems over cells in culture is that aspects such as developmental toxicology and endocrine disruption can be studied, as well as organ specificity of toxicity.

RNAi does, however, have some limitations as a functional screening tool. Two major issues that affect all applications of RNAi are the incomplete knockdown of target genes and off-target effects (the knockdown of genes other than the target). A recently developed functional screening technology that is not affected by these issues uses a human chronic myeloid leukemia (CML) cell line that is haploid for all but one chromosome [85]. Insertional mutagenesis was used to generate a population of cells carrying heterogeneous null mutations of genes. Mutagenized cells can then be cultured in wells of a microplate in the presence of the test compound. Cells with increased resistance to the test compound due to a null mutation form colonies after extended incubation, and the insertion responsible for the mutation can be mapped using a PCR-based technique. This approach has been successfully used to identify host factors used by pathogens [85], and will likely be used extensively in the future for toxicity testing.

To date, functional screens have been conducted using a number of higher eukaryotic systems (Table 2). In human cell systems functional screening has predominantly been used for direct medical applications such as cancer studies, and for some drug development. As use of these technologies becomes more widespread, it is expected that there will be a significant increase in the use of these technologies in toxicology, in order to provide functional data on the roles of human genes in modulating the toxicity of chemical compounds.

## 6. Integrating Functional Data to Identify Toxicity Pathways Will Lead to Comprehensive Cellular Assays

Current high-throughput assays focus on well-characterized cellular response pathways to toxicity. Functional toxicogenomics is a powerful technique that can identify currently unknown or unappreciated genetic modulators of chemical toxicity. Computational approaches such as clustering can identify shared gene and pathway requirements for different toxicants. The functional information can subsequently be integrated with existing annotation and high-throughput data through network representation software such as Cytoscape [60]. Analysis of existing networks can identify key genes in cellular response pathways (“hubs”) to toxicity. Selection of high-throughput assays for use in toxicity testing could then be driven by the capability to assess induction or disruption of these “hubs”. For example, a recent analysis of functional genomic data in yeast identified defects in vesicle tethering complexes as conferring sensitivity to particular toxicants [96]. Similarly, we recently found that genes modulating histone acetylation may play a role in arsenic toxicity [59]. High-throughput assays could be developed to assess effects on both of these processes. In addition to the identification of a role for existing pathways, network inference or reverse engineering methods (that have generally focused on gene expression, but that could be applied to quantitative functional data) could identify novel cellular pathways and processes that are required for both the response to, and the induction of, toxicity.

We suggest that data from functional toxicogenomics studies in yeast and other organisms can be used in conjunction with data from other sources (such as expression profiling of exposed human populations) to provide a “systems biology” approach to studying the toxicity of compounds [97]. Functional toxicogenomics can be used to define both novel and more specific toxicological endpoints, and inform the choice of assays used in high-throughput screening. Functional toxicogenomics is therefore a vital tool in advancing molecular toxicology, forming part of an integrated approach to meet the challenges of 21^st^ century toxicity testing.

## Figures and Tables

**Table 1.** Functional toxicogenomics studies in yeast using Affymetrix TAG microarray platforms.

**Array Platform**	**Compound(s) Tested**	**YKO Collection Used**	**Reference**
TAG3	High salt; sorbitol; galactose; pH8; minimal medium; nystatin	Homozygous	[36]
TAG3	Glycerol; ethanol; lactate; dinitrophenol	Homozygous	[41]
TAG3	Alverine citrate; atorvastatin; methotrexate; 5-fluorouracil (5-FU); miconazole; amphotericin B; lovastatin; cisplatin; itraconazole; fluconazole; dyclonine; fenpropimorph	Heterozygous	[42]
TAG3	None (haploinsufficiency profiling)	Heterozygous	[43]
TAG3	DNA damaging agents	Homozygous	[44]
TAG3	ER stressors (tunicamycin; β-mercaptoethanol)	Homozygous	[45]
TAG3	Chromium	Heterozygous	[46]
TAG3	Neurotoxicants	Homozygous	[47]
TAG3	316 compounds	Homozygous; heterozygous	[48]
TAG3/4*	214 psychoactive drugs	Homozygous; heterozygous	[49]
TAG3	Iron and copper overload	Homozygous	[50]
TAG4	Imidazo[1,2-*a*]pyridines and –Pyrimidines	Homozygous; heterozygous; haploid	[51]
TAG3	Iron deficiency	Homozygous	[52]
TAG4	Sodium arsenite and monomethylarsonous acid (MMA3)	Homozygous	[53]
TAG4	Methyl methanesulfonate (MMS); cisplatin; compound 1561-0023	Homozygous; heterozygous	[54]

* Not specified.

**Table 2.** Selected functional genomics studies in higher organism systems.

**System (organism)**	**Outcome**	**Reference**
shRNA bar code (human)	Identification of genes whose suppression confers resistance to p53-induced growth arrest	[86]
shRNA bar code (human)	Identification of genes whose suppression confers resistance to p53-induced growth arrest	[87]
shRNA bar code (human)	Identification of genes whose suppression confers resistance to Herceptin	[88]
siRNA (human)	Identification of genes required for paclitaxel tolerance	[89]
RNAi (*C. elegans*)	Identification of genes involved in the toxicogenesis of a polychlorinated biphenyl (PCB)	[82]
shRNA (human)	Identification of radiation susceptibility genes	[90]
siRNA (human)	Identification of genes required for stress resistance	[91]
shRNA bar code (human)	Identification of genes required for cancer proliferation	[92]
shRNA (human)	Identification of genes required for induction of apoptosis	[79]
RNAi (zebrafish)	Characterization of novel human platelet proteins	[93]
siRNA (human)	Identification of genes whose suppression confers resistance to epirubicin	[94]
Mutagenized haploid cells (human)	Identification of diptheria toxin pathway genes	[85]
RNAi (*D. melanogaster*)	Identification of modifiers of mutant Huntingtin aggregate formation	[95]
